# Assessing the Impact of Copper and Zinc Oxide Nanoparticles on Soil: A Field Study

**DOI:** 10.1371/journal.pone.0042663

**Published:** 2012-08-08

**Authors:** Daniel Collins, Todd Luxton, Niraj Kumar, Shreya Shah, Virginia K. Walker, Vishal Shah

**Affiliations:** 1 Department of Biology, Dowling College, Oakdale, New York, United States of America; 2 National Risk Management Research Laboratory, U.S. Environmental Protection Agency, Cincinnati, Ohio, United States of America; 3 Department of Biology and School of Environmental Studies, Queen’s University, Kingston, Ontario, Canada; RMIT University, Australia

## Abstract

It is not known if the annual production of tonnes of industrial nanoparticles (NPs) has the potential to impact terrestrial microbial communities, which are so necessary for ecosystem functioning. Here, we have examined the consequences of adding zero valent copper and zinc oxide NPs to soil in pots that were then maintained under field conditions. The fate of these NPs, as well as changes in the microbial communities, was monitored over 162 days. Both NP types traveled through the soil matrix, albeit at differential rates, with Cu NPs retained in the soil matrix at a higher rate compared to ZnO NPs. Leaching of Cu and Zn ions from the parent NPs was also observed as a function of time. Analysis of microbial communities using culture-dependent and independent methods clearly indicated that Cu and ZnO NPs altered the microbial community structure. In particular, two orders of organisms found in rhizosphere, *Flavobacteriales* and *Sphingomonadales*, appeared to be particularly susceptible to the presence of NPs. Together, the migration of NPs through soil matrix and the ability of these potential pollutants to influence the composition of microbial community in this field study, cannot help but raise some environmental concerns.

## Introduction

Engineered metal nanoparticles (NPs) have numerous applications in a variety of consumer goods and industrial processes due to their unique physical, chemical and biological properties. As a consequence, the elevated production to meet this demand will result in an increase in NP release into the environment. To date, there is very little understanding on how such discharged NPs will influence microbial biodiversity. Microorganisms play important roles in geologic, hydrologic and ecological cycles, and any change in microbial diversity can potentially influence environmental quality and health, and even human development [1]–[3]. Previously, the effect of engineered metal NPs on terrestrial microbial communities has been tested under laboratory conditions [4]–[10], but here we have investigated the effect of zinc oxide nanoparticles (ZnO NPs) and zero valent copper nanoparticles (Cu NPs) on the soil microbial community in pots under field conditions.

ZnO NPs are used in electronics, personal care products, biosensors, food additives, pigments and rubber manufacture [11]. Similarly, zero valent Cu NPs are used in electronics, ceramics, films, polymers, inks, metallics, lubricant oil, coatings and health care products [12]. Their prevalence virtually ensures that these NPs may be considered pollutants and indeed, it has been reported that NPs have been detected in waste streams [13]–[14]. In these experiments, an effort has been made to correlate the observed toxicity with the speciation and migration of the NPs through the soil matrix. As a result, we believe that this is the first study to investigate the fate and effect of NPs under field conditions.

## Results and Discussion

### Soil and NP Characterization

The agricultural soil used was rich in total organic carbon (TOC), total Kjeldahl nitrogen (TKN) and phosphorus with values of 13100 mg/kg, 980 mg/kg and 357 mg/kg, respectively. The average soil pH was 7.5±0.2 (n = 5). Low levels of metals including Cu and Zn, as determined by acid digestion, were present in the soil (Table S1). K-edge X-ray Absorption Near Edge Structure (XANES) spectra indicated that Cu^2+^ orZn^2+^ in the untreated soil were adsorbed to a mineral or organic surface. To elucidate this further, reference spectra of Cu and Zn adsorbed to ferrihydrite, alumina γ-Al_2_O_3_, bentonite, and humic acid (Sigma Aldrich, St Louis, MO) were used as analogs for organic matter and mineral phase present in soils and to model the Cu and Zn XANES data using Linear Combination Fitting (LCF) (Figure S1). Results from the LCF analysis indicated that for Cu, 43% was adsorbed to alumina and 57% to humic acid. For Zn, the distribution was 27% ferrihydrite, 32% smectite, and 41% alumina. For both metals, the species present represent Cu and Zn phases that are present in both unperturbed and metal-contaminated soils [15]–[20].

NPs were analyzed using several techniques, with transmission and scanning electron microscope (TEM and SEM) images showing that both the Cu and ZnO NPs were aggregated. Attempts to de-aggregate the material were unsuccessful, and as a result, obtaining the true particle size distribution was not possible. Aggregation also prevented the use of dynamic light scattering as a means of estimating particle size. TEM data obtained for the Cu NPs revealed a wide range of particle sizes (Figure S2 and not shown) with particles generally spherical in shape and sized between <10 and 200 nm. SEM images revealed that the ZnO NPs were elongated (between 15–50 nm in width and 50–20 nm in length) and lacked well-defined crystal faces (Figure S2 and not shown).

**Figure 1:**
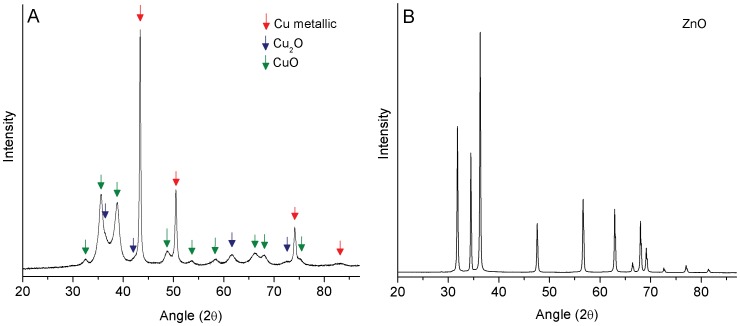
X-ray diffraction patterns for copper (A) and zinc oxide (B) nanoparticles. Different color arrows indicate specific copper phases. Speciation of the Cu phase was determined by comparison with the International Center for Diffraction data (ICDD) powder diffraction files with experimental data (Cu metal *01-085-1326*, Cu_2_O *01-078-2076*, CuO *00-041-0254*). ICDD files for ZnO were *01-079-0206*.

Powdered X-ray diffraction (XRD) (Figure 1) identified three prominent crystalline phases associated with the Cu NPs: metallic Cu, cuprite (Cu_2_O), and tenorite (CuO). Based on X-ray peak intensities the most abundant phase was metallic Cu followed by CuO and Cu_2_O (Figure 1a). The presence of three phases was not unexpected due to the oxidation of Cu on the outer shell and is similar to previously reported results [21]–[22]. Diffraction patterns for the ZnO NPs revealed a single ZnO phase (Figure 1B). NP speciation was also evaluated by LCF analysis of the normalized, normalized derivative, and χ data. For Cu the reference materials included metallic Cu, Cu_2_O, and CuO (Table 1; Figure S3). Consistent with the XRD results, the LCF analysis showed that metallic Cu was the most abundant phase followed by CuO and Cu_2_O (Figure S3). Again, consistent with the XRD analysis, XANES spectra for Zn indicated that ZnO was the only phase present in the NP sample (Figure S4).

**Table 1:** Powdered X-ray diffraction analysis of the Cu NPs, showing the linear combination fit (with R values referring to a general goodness of fit parameter).

Fitting Spectra	Cu metal %	+/− %	Cu_2_O %	+/− %	CuO %	+/− %	Chi^2^	Reduced Chi^2^	R value
**Norm xm(E)**	41.11	0.20	14.17	0.50	44.72	0.20	0.015	3.9*10^−4^	7.9*10^−4^
**Der Norm xm(E)**	36.96	0.51	13.86	0.62	49.18	0.51	0.005	1.4*10^−5^	0.0148
**χ(k)k^2^**	28.73	2.31	20.28	3.43	50.99	2.18	2.81	0.015	0.04
**Average**	35.60		16.10		48.30				

The point of zero charge (PZC) for the Cu NPs from alkalimetric titration was at a pH of 9.4, and close to previous values ([23] and references therein) (Figure S5). Titration of the Cu NP suspension between pH 6-5 resulted in the immediate oxidation of metallic copper in the NPs. XRD data from titration samples collected immediately after suspension showed a color change at pH 5, coincident with the disappearance of metallic Cu and a dramatic increase in the intensity and presence of diffraction peaks associated with cuprite (Figure S6). The PZC for ZnO NPs was 8.7, again close to previous values ([23] and references therein) (Figure S5). The elevated PZC values for both Cu and ZnO NPs indicated that the NPs would possess a positive surface charge in the soil used for the current study, based on the soil pH (7.5).

### Fate of the Nanoparticles

**Figure 2:**
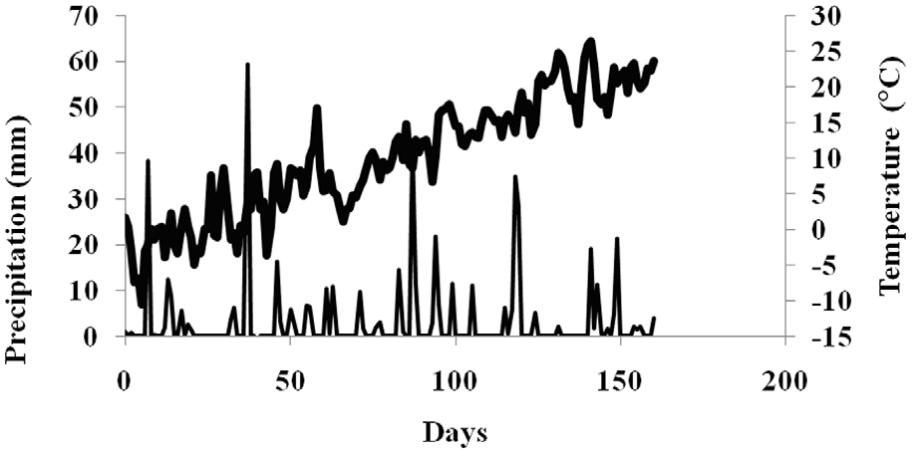
Temperature and precipitation over time at the field study location. Bold and thin lines represent temperature and precipitation, respectively. The parameters were measured using Davis-Vantage Pro2. Day 0 was January 19, 2011.

Cu and ZnO NPs were deposited on the soil surface and allowed to freely migrate through the soil while exposed to environmental conditions over 160 days. Daily temperature and precipitation (total of 495 mm) were monitored over the field study period (Figure 2).

**Figure 3:**
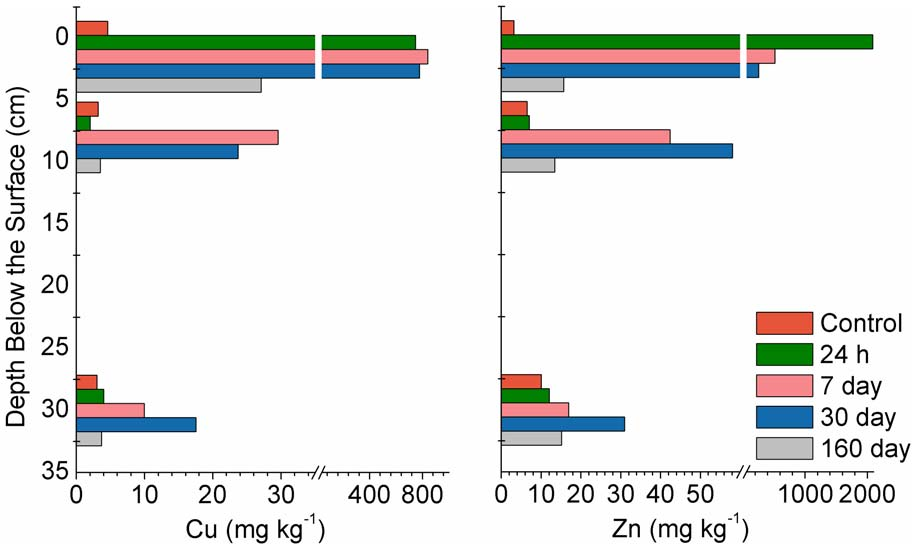
Concentration of Cu and Zn in the extracted soil cores as a function of depth and time. The break in the X-axis occurs at 35 and 60 mg kg^−1^ for Cu and Zn, respectively.

**Figure 4:**
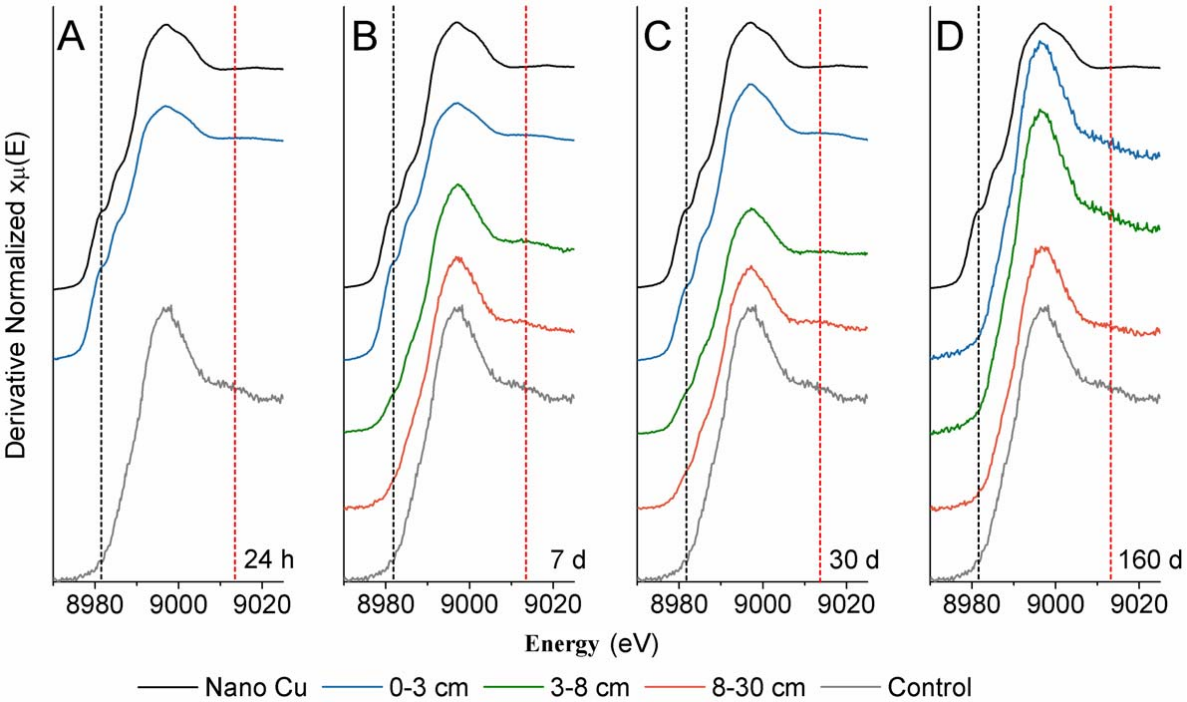
Cu K-edge powdered X-ray diffraction spectra of soil cores at different depths at the four time points. All panels (A–D) show spectra for a Cu NP reference (black line) and the untreated, control soil (grey line). Spectral features are indicated by dashed lines:the Cu pre-edge feature associated with zero valent Cu^0^ (black dashed line) and the post edge feature in the untreated soil,a shoulder associated with adsorbed Cu^2+^ (red dashed line).

**Figure 5:**
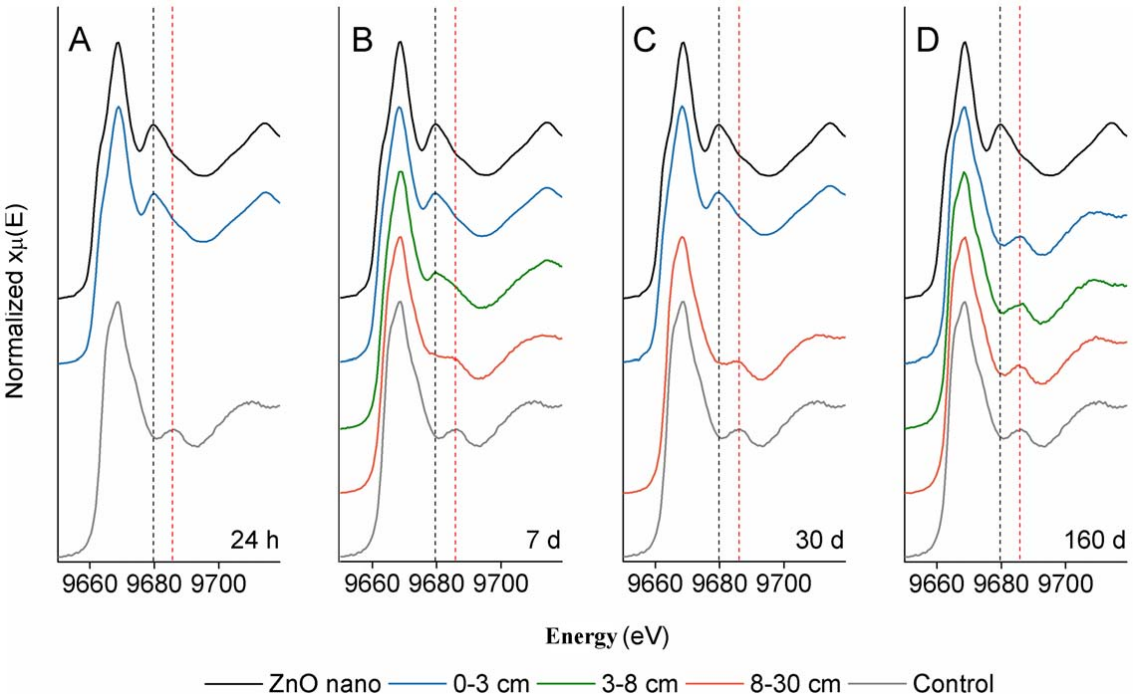
Zinc K-edge powdered X-ray diffraction spectra of soil cores at different depths at the four time points. All panels (A–D) contain a reference spectrum for ZnO NP (black line) and untreated soil (grey line). Spectral features associated either with the ZnO NPs (black dashed lines) or the untreated soil (red dashed lines) are indicated.

The first soil pots were sampled 24 h after NP addition and prior to any precipitation. There was no translocation of the Cu or Zn, and no detectable change in Cu and Zn soil concentrations as a function of depth or NP speciation based on XANES analysis (Figures 3, 4, 5). It should be noted that Cu and Zn K-edge XANES data (Figures 4 and 5, dashed lines) show spectral features unique to the NPs (black dashes) and the Cu and Zn species present in the untreated soil (red dashes). Figure 4 also shows a pre-edge feature indicative of zero valent Cu (Cu^0^; shoulder), and a post-edge feature associated with adsorbed Cu (Cu^2+^; red dashes). Figure 5 demonstrates the XANES spectra post-edge peak indicative of ZnO (black dashes), and a post-edge peak associated with adsorbed Zn (red dashes).

Prior to sampling on day 7, there was a snowfall (total of 2 mm of precipitation). XANES analysis at the surface of the day 7 samples showed that Cu and ZnO NPs were the only phases present, but there was a significant decrease in the Zn concentration and little change in the Cu concentration (Figure 3). Although no NPs were detected in the 2^nd^ horizon at 24 h, by 7 days there was a significant increase in the Cu and Zn concentration at 3–8 cm, and detectable levels in the lower 3^rd^ horizon. For Cu NP treatment pots, the emergence of the Cu^0^ shoulder in the 2^nd^ horizon (Figure 4B, black dashes) provides direct evidence for NP transport in the soil. Further, LCF analysis of the three horizon sections at this sampling period revealed evidence of Cu NPs in all three sections (Table 2). For ZnO NPs, the presence of the post-edge Zn peak at 9679 eV (Figure 5B, black dashed line) indicates that some of these particles had leached into the 2^nd^ horizon. As with the Cu NPs, LCF analysis of all three horizons indicated that ZnO was also present in the 3^rd^-horizon section (Table 2). It is unlikely that the modest amount of melting snow formed large pores in the soil facilitating NP transport in all the experimental pots. Indeed, several studies evaluating ZnO and Cu NP transport in saturated columns have shown that both NPs are mobile [24]–[26] and this mobility is likely enhanced by absorption to organic acids through steric interactions and electrostatic stabilization [24]–[28]. Thus, the high organic carbon content in the agricultural soil likely contributed to an organic surface coating and resulted in NP mobility through the soil. Under our experimental conditions it appears that ZnO is more mobile than Cu based on the decrease in Zn concentration in the surface layer and the coincident increase of Zn in the 2^nd^ horizon (Figure 3). When these results are combined with those reported in the literature, it suggests that metallic Cu NPs can be considered the least mobile NPs when compared to Fe_3_O_4_, CuO, TiO_2_ and ZnO NPs [24].

**Table 2:** K-edge X-ray absorption near-edge structure (XANES) spectral results (linear combination fit analysis) showing normalized Cu and Zn XANES data with Cu and Zn speciation are presented as a function of time and depth (corresponding to the spectral results in Figures 3 and 4).

		Cu species and NPs			Zn species and NPs			
Day	Depth	Cu alumina %	Cu humic %	Cu NP %	Zn alumina %	Zn ferrihydrite %	Zn smectite %	ZnO NP %
	0–3	0	0	100	0	0	0	100
1	3–8	–	–	–	–	–	–	–
	8–30	–	–	–	–	–	–	–
	0–3	0	0	100	0	0	0	100
7	3–8	31	12	57	7	0	40	53
	8–30	10	54	36	18	0	59	24
	0–3	0	0	100	0	0	0	100
30	3–8	10	25	66	–	–	–	–
	8–30	5	43	52	25	11	57	7
	0–3	22	69	9	36	4	60	0
160	3–8	8	74	18	38	2	61	0
	8–30	32	51	17	36	7	57	0

In addition to the migration of NPs, dissolution of Cu and ZnO NPs was also apparent after 7 days. The 9-fold increase in Cu and Zn concentration in the 2^nd^-horizon was partially due to translocation. However, since the Cu and ZnO NPs only accounted for 50% of the Cu and Zn species identified (Figures 3, 4B, 5B, Table 2), it appears that the NPs in the 1^st^ horizon released Cu and Zn ions that then migrated downwards (Figures 3, 4B, 5B, Table 2). The same conclusion can be made for Cu and Zn in the 3^rd^ horizon, based on the ∼2.5-fold increase in metal concentration with ∼40 and 25% of the Cu and Zn species, respectively, present in the NP form (Figures 3, 4B, 5B, Table 2).

The study site received significant precipitation in the form of rain and snow in the 7–30 day period (Figure 2). Nevertheless, there was no significant difference in the concentration or speciation of Cu and Zn in the surface layer and only modest differences in the 2^nd^ horizon at the third sampling period, compared to the 7 day samples (Figure 3). In the 3^rd^ horizon, however, the concentration of Zn and Cu doubled. This significant increase in Cu NPs in the 3^rd^ horizon was further evidenced by the emergence of the Cu^0^ pre-edge feature (Figure 4C, black dashes; Table 2), and clearly demonstrated the continued leaching of these NPs. Zinc speciation in the 3^rd^ horizon indicated significant dissolution of the ZnO (Table 2) and was corroborated by the strong post-edge feature (Figure 5C, red dashes), associated with an adsorbed Zn phase. It has been reported that there is a rapid dissolution/transformation of ZnO to a Zn adsorbed phase (pH 7 kaolin suspension) within 24 h [29], and therefore, the dissolution of ZnO NP in soil over a 30 day period would not be unexpected. The 30-day samples also provided additional evidence for the increased mobility of ZnO NPs compared to Cu NPs since there was a marked increase in Zn concentrations throughout the soil profile as compared to Cu. The persistence and decreased mobility of Cu NPs may be a cause of concern with respect to their potential ecological impact on soil surfaces.

After 160 days with increased temperatures and much precipitation (Figure 2), including 10 mm of rain in a 24 h period, the concentration of Zn in the soil had returned to levels close to that of untreated soil (Figure 3). XANES analysis showed that some Cu NP remained but there was no significant presence of the original ZnO NPs. The observation of mobile Cu and Zn species at this time point (Figures 4D; 5D; Table 2), suggests that desorption of the adsorbed metals could be related to the increased temperature.

Less than 0.1% of the original NPs were recovered in the soils. There is no evidence in the literature suggesting that these NPs are resistant to microwave-assisted acid extraction and strict quality assurance and control procedures (including, and not limited to blank, spiked, and numerous controls), gave no indication that recovered NP mass was associated with instrument error. Our soil-nanoparticle experimental pots were open systems, and no attempt was made to collect the leachate or prevent the erosion of nanoparticles caused by wind, which would be necessary for determining the relationship between NPs and soil on a mass basis. In the future, consideration must be given to controlling wind erosion as well as the collection of soil leachate. Not withstanding these caveats, the analyses presented here clearly demonstrate that both Cu and ZnO NPs are mobile in agricultural soils and have the potential to escape beyond an initial ‘spill’ site.

### Microbial Community Analysis

The impact of Cu and ZnO NPs on the soil microbial community was measured using culture-idependent (Biolog® ecoplates) and culture-independent methods (FAME analysis and pyrosequencing, respectively). Physiological profiles obtained using Biolog® ecoplates showed that NP additions had an immediate impact on the culturable microorganisms (Table 3). Substrate richness (S) signifies the number of substrates used appreciably (optical density>25) by the community and it is a good indicator of the functional diversity within soil [30]. In the top horizon, the control soil had an S value of 22 but this value dropped to 2 (representing only pyruvic acid methyl ester and L-aspargine substrates) right after the addition of Cu NPs. Likewise, the S value dropped to 11 immediately following the addition of ZnO NPs to the soil. Parallel to these decreases in substrate utlization, a decrease in the Shannon diversity index (H) was observed for both the NPs. Since these physiological profiles were done *in vitro*, the added NPs would have been carried over with the soil samples explaining the observed high toxicity, as has been previously reported for Cu and ZnO NPs [12], [28]–[30]. Indeed, the S and H values (Table 3) are inversely related the NP concentration (Figure 3). We therefore suggest that direct contact [31]–[34] between NPs and microbes, such that is achieved in the *in vitro* physiological assays, may be necessary for acute bacterial toxicity. A previous study showed that when Cu NPs were homogenously mixed with the NPs and incubated under laboratory conditions only marginal changes in the microbial community were observed [4]. In this case, we speculate that very few of the microbes were in direct contact with the NPs.

**Table 3:** Microbial community distribution in the soil exposed to copper or zinc oxide nanoparticles under field conditions as measured using FAME analysis and microbial community diversity indicators as measured using community level physiological profile analysis.

LAYER	EXPERIMENTAL SAMPLE	FAME ANALYSIS								BIOLOG ANALYSIS		
		G+	G−	Diatoms	Eukaryote	Fungi	Methanobacter	Actinomycetes	Anaerobic	H	S	E
**TOP**	**Control- 0 day**	30	36	16	2	4	1	11	0	3.0	22	2.2
	**Copper- 0 day**	29	36	18	2	4	1	10	0	2.2	2	7.3
	**Zinc- 0 day**	30	37	17	2	4	1	9	0	2.5	11	2.4
	**Control- 7 days**	30	37	17	2	3	1	11	0	2.5	14	2.2
	**Copper- 7 days**	29	37	17	2	4	1	10	0	2.1	3	4.4
	**Zinc- 7 days**	29	37	17	3	4	1	8	0	2.1	5	3.0
	**Control- 30 days**	29	37	17	2	4	0	11	0	2.8	17	2.2
	**Copper- 30 days**	29	38	16	1	3	1	12	0	0.0	0	0.0
	**Zinc- 30 days**	28	37	16	2	4	2	11	0	2.7	15	2.3
	**Control- 160 days**	24	39	19	2	3	1	12	0	2.7	16	2.2
	**Copper- 160 days**	25	39	17	2	3	1	12	0	2.3	8	2.5
	**Zinc- 160 days** *									2.4	12	2.3
**MIDDLE**	**Control- 0 day**	31	35	16	2	4	1	10	0	3.0	24	2.1
	**Copper- 0 day**	31	35	16	2	3	1	11	0	3.1	24	2.4
	**Zinc- 0 day**	30	35	17	2	5	1	10	0	2.9	20	2.2
	**Control- 7 days**	31	37	16	2	3	1	10	0	2.8	15	2.3
	**Copper- 7 days**	31	35	16	2	4	1	10	0	2.9	20	2.2
	**Zinc- 7 days**	29	36	17	2	4	1	11	0	2.9	18	2.3
	**Control- 30 days**	28	36	17	3	4	1	10	0	2.8	18	2.3
	**Copper- 30 days**	28	38	16	1	3	1	13	0	2.6	14	2.2
	**Zinc- 30 days**	28	38	17	2	4	0	12	0	2.7	15	2.2
	**Control- 160 days**	25	40	18	2	3	2	12	0	2.4	11	2.3
	**Copper- 160 days**	25	40	16	2	3	2	13	0	2.4	12	2.3
	**Zinc- 160 days**	24	40	18	2	3	2	12	0	2.4	11	2.3
**BOTTOM**	**Control- 0 day**	32	35	16	2	5	1	10	0	2.9	17	2.3
	**Copper- 0 day**	30	36	16	2	4	1	11	0	3.0	21	2.4
	**Zinc- 0 day**	30	36	17	2	4	1	10	0	3.0	23	2.3
	**Control- 7 days**	29	37	16	2	4	1	10	0	2.7	14	2.2
	**Copper- 7 days**	28	36	17	3	6	0	10	0	2.8	18	2.3
	**Zinc- 7 days**	28	37	17	2	5	2	10	0	2.8	16	2.2
	**Control- 30 days**	29	36	17	2	4	1	11	0	2.7	15	2.5
	**Copper- 30 days**	29	37	15	1	3	1	13	0	2.4	9	2.2
	**Zinc- 30 days**	29	37	17	2	4	1	11	0	2.8	19	2.2
	**Control- 160 days**	26	40	17	2	3	2	11	0	2.3	12	2.3
	**Copper- 160 days**	25	40	16	2	3	2	13	0	2.3	11	2.2
	**Zinc- 160 days**	25	41	16	2	3	2	11	0	2.3	12	2.2

* FAME data for top layer zinc 160 days not obtained due to contamination of sample.

Culture-independent FAME analysis showed no significant impact of the addition of either NP on the overall microbial soil profile across all three horizons and over time. Gram-negative bacteria were more prevalent (65%) than Gram-positive bacteria in control soil and this proportion remained the same throughout the horizons at all time points with minor changes irrespective of the NP treatment. Likewise, diatom and actinomycete fatty acid signatures showed no impact due to neither the NPs nor the metallic ion species.

**Figure 6:**
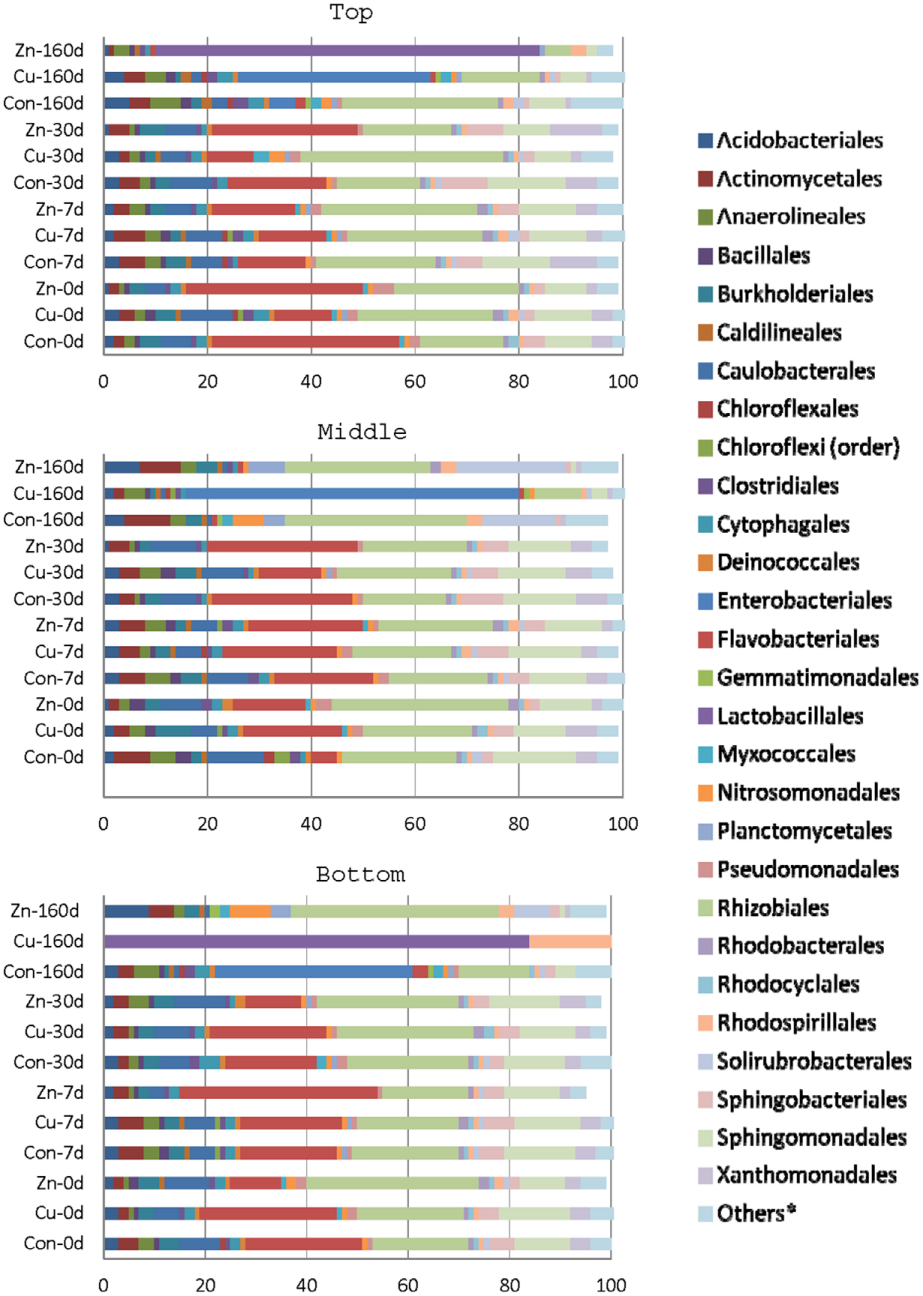
Relative abundance of bacterial orders (%) determined by pyrosequencingin the top, middle and bottom horizons of unexposed and nanoparticle-exposed soils. Horizontal colored bars represent different bacterial orders identified in soils from control (Con), Cu NP (Cu) and Zn NP (Zn) experimental pots treated for 160, 30, 7 and 0 days. *Others represents all of the orders in which the percentage was <1.

While physiological profiles clearly showed microbial toxicity, likely due to the direct contact with the NPs, FAME analysis indicated that the proportion of major bacterial types did not change after NP exposure. Thus, it was still not clear if the overall community composition significantly altered under these field conditions. Therefore, we additionally surveyed the bacterial community through pyrosequencing having first treated the soil so as to decrease the proportion of DNA derived from dead or dying cells, a method that is not applicable prior to FAME analysis. Although DNA community analysis is the best means of evaluating soil changes, caution needs to be exercised when using such a responsive technique in field experiments since microbial flora can be unevenly distributed, with variation introduced by eukaryotes, including invertebrates, birds, plants and mammals. Indeed, the higher percentages of *Enterobacteriales* and *Lactobacillales* in some of the samples (Figure 6, Table S2) can likely be attributed to the bird feces [35]–[38]. Despite such perturbations, however, an overall impact of the microbial community by the introduced NPs was evident (Table 4).

**Table 4:** % of Flavobacteriales, Rhizobiales and Sphingomonadales in control soil and that exposed to nanoparticles. (Con, Control; Cu, Cu nanoparticles; Zn, ZnO nanoparticles).

	Con-0d	Cu-0d	Zn-0d	Con-7d	Cu-7d	Zn-7d	Con-30d	Cu-30d	Zn-30d	Con-160d	Cu-160d	Zn-160d
**Top horizon**												
Flavobacteriales	60%	23%	52%	26%	25%	28%	38%	16%	52%	6%	5%	9%
Rhizobiales	26%	54%	36%	47%	53%	53%	33%	71%	32%	77%	70%	70%
Sphingomonadales	14%	23%	12%	27%	22%	19%	29%	14%	17%	18%	25%	21%
**Mid horizon**												
Flavobacteriales	11%	38%	24%	39%	41%	40%	47%	25%	48%	2%	6%	2%
Rhizobiales	51%	42%	58%	39%	35%	40%	29%	47%	32%	97%	72%	94%
Sphingomonadales	38%	21%	18%	22%	25%	20%	24%	28%	20%	1%	22%	4%
**Bottom horizon**												
Flavobacteriales	43%	44%	19%	35%	38%	58%	33%	38%	20%	14%	0%	1%
Rhizobiales	36%	34%	64%	38%	38%	25%	45%	44%	53%	65%	100%	98%
Sphingomonadales	21%	23%	18%	26%	24%	17%	22%	18%	26%	21%	0%	1%

Control soil samples showed that members of the *Rhizobiales*, *Flavobacteriales* and *Sphingomonadales* orders formed the majority within the bacterial community (Figure 6, Table S2), as would be expected in agricultural soils [39]. All of these organisms are found in high numbers in the rhizosphere [40]. Viable cells are unlikely to be introduced by vertebrate excreta and thus any shift in the ratio of members of these orders should be suitable for the evaluation of NP toxicity. The ratio of these bacterial orders in the three horizons as a function of time and treatment is shown in Table 4. When comparing the ratio in control *vs* NP-treated samples for the top horizon at time 0, one can infer that representatives of *Rhizobiales* are not immediately impacted by Cu or ZnO NPs. In contrast, bacteria belonging to *Flavobacteriales* appeared to be highly susceptible to Cu NPs, if not to ZnO NPs (Table 4). As the NPs were translocated towards the lower horizons as a function of time (Figure 3), the concordant decrease in *Flavobacteriales* is evident in these horizons. The majority of the identified genera representing this order are mesophiles and thus more metabolically active at moderate temperatures. As a result, it would have been active bacteria that were exposed to the NPs/metallic ion species as they travelled to lower horizons while the soil warmed to summer conditions, and active microbes appear to be more susceptible to NP toxicity [4], [41]. In fact, there was almost a complete absence of bacteria belonging to both *Flavobacteriales* and *Sphingomonadales* in the 3^rd^ horizon at 160 days. Taken together, the pyrosequencing data validates the hypothesis that exposure to NPs significantly alters the microbial community structure, similar to the results obtained by laboratory studies using metal NPs [4], [8]. It should be recalled that at 160 days, almost no original NPs were detected in this horizon (Figure 3), suggesting that toxicity had either occurred prior to that time or that leachate containing ion species affected the consortia. The low sensitivity of *Rhizobiales* to Cu and ZnO NPs is distinct from a previous study [4] that showed susceptibility to 0.067% Ag NPs. It is possible that the differential sensitivity of *Rhizobiales* to types of metal NPs could be attributed to different mechanism of action for individual NPs. Further studies need to be carried out to test the hypothesis. Based on this first field study of the effects of Cu and ZnO NPs on soils, we can conclude that they migrated through the soil matrix as NPs, and the leaching of ions from the NPs occured parallel to that of particle transport. We have been careful not only to investigate the effects of metallic NPs on soil chemistry and biota but have also considered the combined effect of NPs and ions when evaluating the results. The microbial community is indeed altered by the NPs/metal ions as they migrate through the matrix. While it is too soon to correlate these structural changes in the community to functional changes in the ecosystem, considering that we are manufacturing tonnes of NPs yearly [42], we believe that immediate attention is warranted to understand this correlation and to study the overall impact of NPs on biogeochemical cycles.

## Materials and Methods

### Nanoparticle Characterization

Zero valent copper and zinc oxide NPs were purchased from Sun Innovations (Fremont, CA) and extensively characterized prior to use. NP crystal structure was determined by powder XRD using a PANalyticalXpert pro MPD (Westborough, MA) with CuKα radiation and a scan rate of 0.02° θ, from 10 to 85° 2θ. Six scans were collected and averaged prior to analysis. The PZC for Cu and ZnO was determined by measuring the point of zero net proton charge in a NaCl background electrolyte [43]. Titrations were carried out from pH 10−4.5 and 10−6.5 for Cu and ZnO, respectively. The oxidation state and local bonding environment of Cu and ZnO NPs were examined using X-ray absorption fine structure (XAFS) spectroscopy. The K-edge spectra were collected at beam line 10-BM (Materials Research Collaborative Access Team, Advanced Photon Source, Argonne National Laboratory, Argonne IL). NPs were diluted with polyvinylpyrrolidone (PVP) to a final concentration of 0.05%. The diluted samples were then compressed into pellets using a hand press and sealed between two strips of Kapton tape prior to analysis. Adsorption spectra were collected at the K-edge energies of 8979 and 9659 eV, respectively. Data collection was done in fluorescence mode using a 4-element solid-state Si-detector. The synchrotron was operated at 7.0 GeV at a nominal 100 mA fill current. The energy of a Si (111) double crystal monochromator was calibrated using an elemental Cu and Zn foil. Scans were collected from 8779–9979 eV and 9459–10659 eV for Cu and Zn, respectively. All spectra were collected at room temperature at ambient pressure with a minimum of three scans (and up to 5) collected for each sample. Particle size and shape were determined by field emission scanning electron microscopy (FESEM) (JEOL JSM-7000Scanning Electron Microscope, Peabody, MA), and scanning transmission electron microscopy (STEM)(JEOL JEM-2100F Transmission Electron Microscope, Peabody, MA). Samples for TEM and SEM analysis were prepared by evaporation of a dilute ethanol suspension. A 0.1 or 3 µL droplet was deposited onto a nickel mesh TEM grid or carbon tape, respectively and dried at 80°C for 1 h. The SEM operated at operated at 15 kV with a 2 nm resolution and the TEM was operated at 200 kV with a resolution of 0.1 nm. Nanoparticle characterization experiments were carried out in triplicate and the results represent an average of three measurements.

### Soil Characterization

The soil used for the field study was obtained from an agricultural soil-distribution center (New York State; 40.76° N, 73.27° W), from a single mound of topsoil. When the experiments were initiated, the first 15–16 cm of the soil was frozen, and thus soil was taken from deeper in the mound. The soil was transported in plastic bags and any visible debris (plant matter, rocks, and wood chips) was removed manually before soil was added to the pots. A portion of the soil was sampled to determine the pH by measuring the supernatant of a 1∶1 soil to Milli-Q water mixture. Similarly, the chemical composition of the soil was determined by inductively coupled plasma optical emission spectroscopy (ICP-OES) after carrying out microwave assisted nitric/hydrochloric acid digestion [44]. Briefly, a 0.2–0.5 g sample of the homogenized, dried soil was extracted via microwave-assisted digestion and chemical extraction using boiling nitric and hydrochloric acid. The resulting extracts were analyzed by inductively coupled plasma optical emission spectroscopy (ICP-OES; Thermo Elemental IRIS Intrepid; Madison, WI). The accuracy (±5–7.5%) and precision (±3–12% depending on the element) of the instrument were verified before and after analysis. TOC, TKN and total phosphorus in the soil were analyzed commercially (Long Island Analytical Laboratories, New York) using EPA 9060, ASTM D3590-89 & 02(A) and SM 18–21 4500 – PE methods, respectively. pH and chemical composition of the soil were determined three times and averaged.

### Soil Treatments

Plastic planting pots (23.6 cm×26.7 cm, Ames True Temper) containing a rolled rim and a saucer at the bottom were filled with soil to a height of 20.3 cm and transported to the field site (located close to the soil originating point at 40.72° N, 73.09° W). The area was secured to prevent unauthorized access. No specific permits were required from any agencies as the contaminated soil was never directly introduced into the environment. In addition, as Dowling College houses The Center for Estuarine, Environmental, and Coastal Oceans Monitoring (CEECOM) at this location, no specific permission was required to place the pots at this location. The field study did not involve endangered or protected species. After transport, the soil was allowed to stabilize for 7 days prior to adding the Cu or ZnO NPs (550 mg; starting on Jan. 19, 2011) by sprinkling over a 5 cm diameter at the center of the soil surface. Control pots received no NPs. At specified times (see results) sample pots were transported to the lab, and a ‘core sample’ was taken from the center by inserting a poly vinyl chloride (PVC) pipe (5 cm diameter). The cored soil sample was separated based on the depth with the top horizon (0−2.5 cm), 2^nd^ horizon (2.5–7.5 cm) and 3^rd^ horizon (7.5–20 cm). The soil from each horizon was mixed for 5 min to decrease soil heterogeneity. One pot each of control, Cu NPs, and ZnO NPs were removed at each time point. Time points were chosen as follows: short term incubation within a month of NP inoculation for the analysis of acute, local effects (1, 7 and 30 days), and long-term incubation (160 days) for long-term transport investigation. Weather data were recorded using Davis-Vantage Pro2 (Product #6312, Model #6152). The instrument was located no more than 2 m from the pots, ensuring it was not directly over them.

### Solid Phase Analysis

After sampling, samples were freeze dried (Millrock Technology, Bench Top Model, Kingston, NY), fractionated based on particle size (2 mm−250 µm, 250–75 µm, and <75 µm) and stored in airtight containers prior to analysis. The concentration of Zn and Cu in each of the soil particle size fractions was determined by microwave-assisted acid digestion using the previously described methods.

The oxidation state and local bonding environment of the NPs in the soil were examined using XAFS spectroscopy as described except that PVP was not added. K-edge spectra were collected for the <75 µm NP fraction. In addition to the soil samples, mineral reference compounds were analyzed. Reference compounds included: metallic Cu, Cu_2_O, CuO, CuCO_3_, aqueous Cu^2+^, CuCl_2_, metallic Zn, ZnO, ZnCO_3_, aqueous Zn^2+^, and ZnCl_2_. Reference scans were also collected for Cu and Zn adsorbed to ferrihydrite, γAl_2_O_3_, smectite, birnesite, and humic acid. Initial Cu and Zn concentrations were 500 mg L^−1^ and conducted at pH 6, in 0.01 M NaCl, and a suspension density of 2 g L^−1^.

XAFS data processing and analysis were conducted using the Athena software package in the IFEFFIT computer program [45]. The collected fluorescence data were averaged and normalized to the edge jump height. The K-edge inflection point was determined using the energy at the maximum in the first derivative of the normalized spectra. Cu and Zn speciation in the soils was determined by linear combination fits utilizing a least squares procedure to fit the reference compounds to the sample spectra. During the fitting procedure, the energy shift of the reference compounds was constrained to ±0.5 eV of the K-edge inflection point. The number of reference compounds to describe a soil samples was kept to a minimum and any reference compound returned with <∼10% or negative concentrations was omitted from the fitting process.

### Microbial CLPP and FAME Analysis

Microbial community physiological profiles were obtained in triplicate using Biolog® Ecoplates (Biolog®, Hayward, CA) using previously established methods [8]. The average of three absorption readings was employed and the values of control wells were subtracted. Occasional negative values were set to zero. Evenness (E) as well as S and H values were calculated as described [5].

Fatty acids were extracted from soil samples and analyzed by Microbial ID (Newark, DE).

### DNA Isolation and 454-pyrosequencing

Soil samples (0.5 g) were first treated with ethidiummonoazide (EMA) as described [46] in order to preferentially amplify DNA from viable cells. DNA was extracted from soil (0.25 g) using Power Soil™ DNA isolation kits (MO BIO Laboratories Inc, Carlsbad, CA). DNA extraction was carried out in duplicate and the samples pooled prior to pyrosequencing. Bacterial tag-encoded FLX ampliconpyrosequencing (bTEFAP) was performed as described previously using the following bacterial primers: Gray28F 5′TTTGATCNTGGCTCAG and Gray519r 5′ GTNTTACNGCGGCKGCTG [47]. One-step PCR (30 cycles) was conducted using a mixture of Hot Start and HotStar (QIAGEN, CA, USA) high fidelity Taq polymerases. Amplicons originating and extending from the bacterial 28F primer were used for initial generation of the sequencing library. Tag-encoded FLX amplicon pyrosequencing analyses utilized a Roche 454 FLX instrument with titanium reagents and procedures were performed at the Research and Testing Laboratory (Lubbock, TX) based upon their protocols (www.researchandtesting.com). Subsequently, all failed sequence reads, low quality sequence ends, tags and primers were removed and any non-bacterial ribosomal RNA (rRNA) sequences and chimeras were removed using B2C2 [48] as previously described [47]. To curate the short reads (<150bp), sequences with ambiguous base calls and sequences with homopolymers>6bp>6bp were removed. To determine the identity of bacteria in the curated data, sequences were denoised, assembled into operational taxonomic units (OTUs) at 96.5% identity, and queried using a distributed. NET algorithm via Blastn+ (KrakenBLAST www.krakenblast.com) against the database of high quality 16S rRNA bacterial sequences. Using a. NET and C# analysis pipeline, the resulting outputs were compiled and data reduction analysis performed as described previously [49].

Based upon the sequence identity determined as the percent of the total length query sequence, aligned with a known sequence in the database, each sequence was assigned to the appropriate taxonomic level based upon the following criteria. Sequences with identity scores, to known or well characterized 16S rRNA sequences, greater than 97% identity were resolved at the species level, between 95–97% at the genus level, between 90–95% to the family and between 85–90% to the order level 80–85% to class and 77–80% to phyla. The percentage of sequences at each bacterial level were then used to determine the relative abundance within and among the individual samples based upon relative numbers of reads within each. Evaluations presented at each taxonomic level, including percentage compilations represent all sequences resolved to their primary identification or their closest relative [48].

## Supporting Information

Figure S1 Cu (A) and Zn (B) K-edge XANES spectra for the untreated soil and Cu and Zn adsorbed to reference materials. The dashed red line represents the LCF best fit. For Cu the LCF results were 43% Cu adsorbed to alumina and 57% Cu adsorbed to humic Acid. For Zn the LCF results were 27% Zn adsorbed to ferrihydrite, 32% Zn adsorbed to smectite, and 41% Zn adsorbed to alumina.
(TIFF)

Figure S2 Visualization of the NPs showing A) a representative TEM micrograph of Cu NPs B) a representative SEM image of ZnO NPs.
(TIFF)

Figure S3 Linear combination fitting results for Cu K-edge A) normalized XANES spectra, B) normalized 1^st^ derivative XANES spectra, and C) extended X-ray Adsorption Fine Structure (EXAFS) χ(k)k^2^ data. Relative percentage values are presented in Table 1.
(TIFF)

Figure S4 **Normalized and the first derivative of the normalized XANES spectra for ZnO nanoparticles, compared to ZnO and ZnCO_3_ reference materials.** Dashed grey lines in the figures highlight the energies corresponding to spectral features present in the ZnO NPs.
(TIFF)

Figure S5 **Alkalimetric titration curves for Cu and ZnO nanoparticles.** The dashed lines indicate the point of zero charge. The grey-circled area indicates the pH region where metallic Cu is oxidized to Cu^1+^ and Cu^2+^.
(TIFF)

Figure S6 **X-ray diffraction pattern of pre- and post- titration Cu nanoparticles.** Different colored arrows indicate specific Cu phases. A break in the Y-axis occurs at an arbitrary value and was used to illustrate changes in the finer features of the XRD pattern.
(TIFF)

Table S1 Elemental composition of the untreated soil determined by EPA Method 3051, at horizons 1–3 (0–3, 3–8 and 8–30 cm, respectively), expressed as mg/kg showing the relative standard deviation (RSD).
(XLS)

Table S2 Bacterial population present in the soil classified at order level. (Con, Control; Cu, Cu nanoparticles; Zn, ZnO nanoparticles).
(XLS)
