# Supplementary figures and images for: Assessing the Impact of Copper and Zinc Oxide Nanoparticles on Soil: A Field Study
Source: PLoS One. 2012 Aug 8;7(8):e42663. doi: 10.1371/journal.pone.0042663 (PMC3414451; doi:10.1371/journal.pone.0042663)

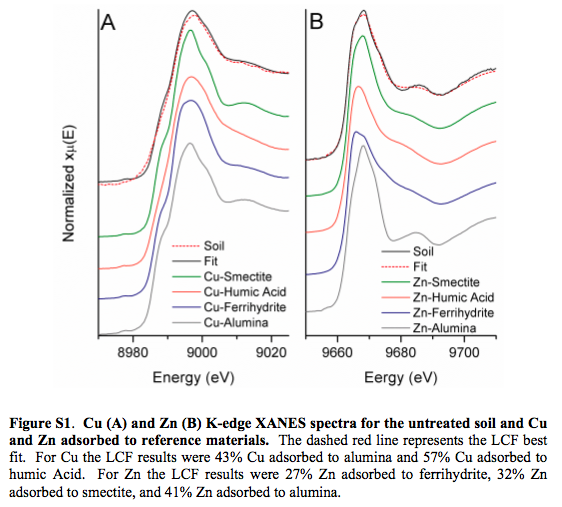

Supplement: Figure S1 — Cu (A) and Zn (B) K-edge XANES spectra for the untreated soil and Cu and Zn adsorbed to reference materials. The dashed red line represents the LCF best fit. For Cu the LCF results were 43% Cu adsorbed to alumina and 57% Cu adsorbed to humic Acid. For Zn the LCF results were 27% Zn adsorbed to ferrihydrite, 32% Zn adsorbed to smectite, and 41% Zn adsorbed to alumina. (TIFF) [file pone.0042663.s001.tiff]

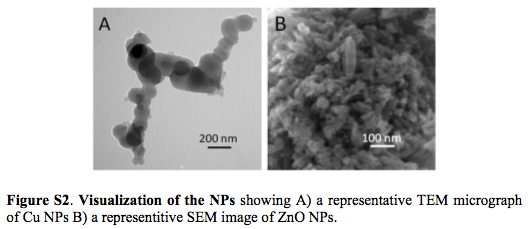

Supplement: Figure S2 — Visualization of the NPs showing A) a representative TEM micrograph of Cu NPs B) a representative SEM image of ZnO NPs. (TIFF) [file pone.0042663.s002.tiff]

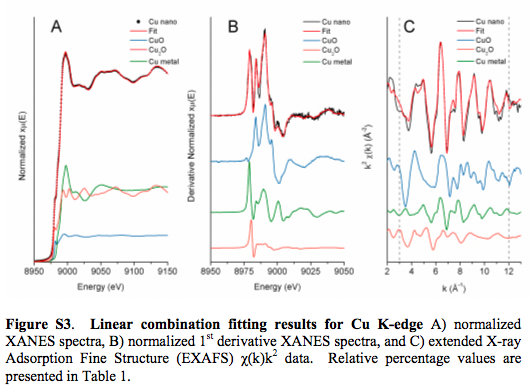

Supplement: Figure S3 — Linear combination fitting results for Cu K-edge A) normalized XANES spectra, B) normalized 1st derivative XANES spectra, and C) extended X-ray Adsorption Fine Structure (EXAFS) χ(k)k2 data. Relative percentage values are presented in Table 1. (TIFF) [file pone.0042663.s003.tiff]

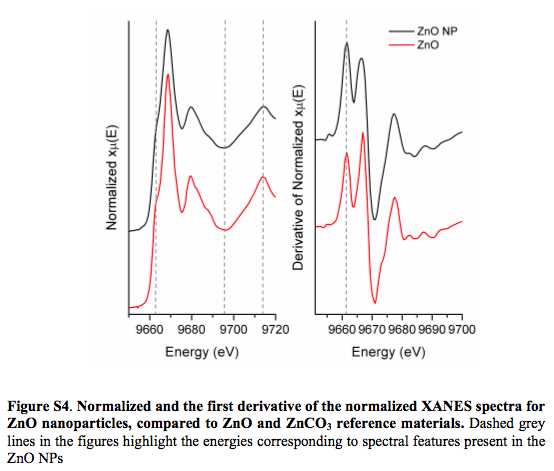

Supplement: Figure S4 — Normalized and the first derivative of the normalized XANES spectra for ZnO nanoparticles, compared to ZnO and ZnCO3 reference materials. Dashed grey lines in the figures highlight the energies corresponding to spectral features present in the ZnO NPs. (TIFF) [file pone.0042663.s004.tiff]

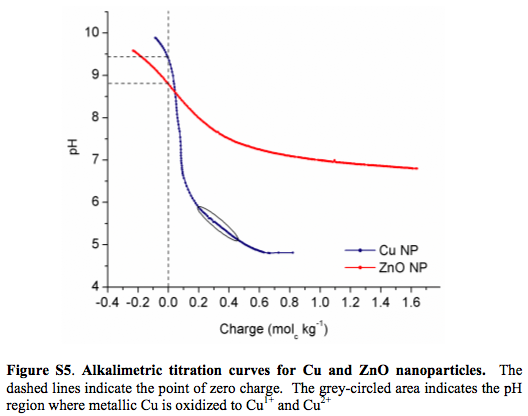

Supplement: Figure S5 — Alkalimetric titration curves for Cu and ZnO nanoparticles. The dashed lines indicate the point of zero charge. The grey-circled area indicates the pH region where metallic Cu is oxidized to Cu1+ and Cu2+. (TIFF) [file pone.0042663.s005.tiff]

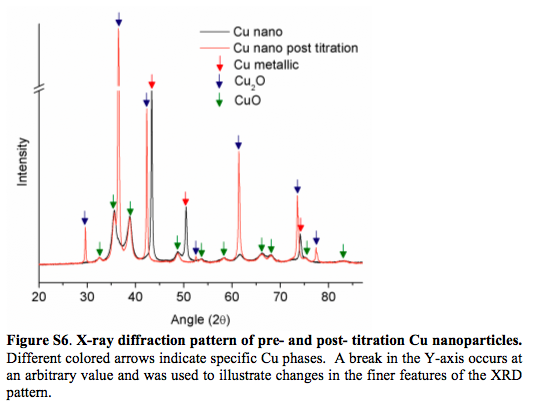

Supplement: Figure S6 — X-ray diffraction pattern of pre- and post- titration Cu nanoparticles. Different colored arrows indicate specific Cu phases. A break in the Y-axis occurs at an arbitrary value and was used to illustrate changes in the finer features of the XRD pattern. (TIFF) [file pone.0042663.s006.tiff]
